# ZIKV infection causes placental inflammation through activating PANoptosis

**DOI:** 10.1128/jvi.01759-25

**Published:** 2025-11-20

**Authors:** Shuqing Zhang, Delin Chen, Kexin Zhang, Minjie Liu, Hao Liang, Linyue Liang, Jingyao Liang, Minqi Liang, Shu An, Mingcui Lyu, Junying Zhu, Shangwei Li, Dingwen Hu, Xun Zhu, Jueheng Wu, Zhenjian He, Mengfeng Li

**Affiliations:** 1 School of Basic Medical Sciences, Southern Medical University, Guangzhou, China; 2 Clinical Experimental Center, Jiangmen Engineering Technology Research Center of Clinical Biobank and Translational Research, Jiangmen Key Laboratory of Precision and Clinical Translation Medicine, Jiangmen Central Hospital, Jiangmen, China; 3 Department of Microbiology, Zhongshan School of Medicine, Sun Yat-sen University 626118 , Guangzhou, China; 4 School of Public Health, Sun Yat-sen University 71023 , Guangzhou, China; 5 Department of Laboratory Medicine, The First Affiliated Hospital of Sun Yat-sen University 625771 , Guangzhou, China; 6 Key Laboratory of Tropical Disease Control, Ministry of Education, Sun Yat-sen University 26469 , Guangzhou, China; The Ohio State University, Columbus, Ohio, USA

**Keywords:** ZIKV, PANoptosis, placental inflammation, trophoblast cells

## Abstract

Infection by the Zika virus (ZIKV) during pregnancy can cause congenital Zika syndrome (CZS) or other central nervous system conditions in infants, underscoring the importance of understanding the role of ZIKV-induced placental damage in the development of CZS. Here, we established a ZIKV-infected pregnant mouse model and examined the pathological changes in the placenta following ZIKV infection. We found that ZIKV infection induces severe placental inflammation associated with trophoblast PANoptosis. Specifically, ZIKV activates RIG-I and recruits ASC, caspase-1, NLRP3, caspase-8, and RIPK1 to form a PANoptosome complex, leading to the activation of PANoptosis. Moreover, the combined use of Z-VAD-FMK with GSK872 or with the RIG-I inhibitor RIG012 robustly suppressed ZIKV-induced cell death, attenuated the inflammatory response in trophoblast cells and the placenta induced by ZIKV infection. Collectively, we elucidate a previously unrecognized mechanism by which ZIKV infection causes severe placental inflammation by activating PANoptosis and provide a foundation for the potential application of anti-PANoptotic therapy against ZIKV-associated diseases.

**IMPORTANCE:**

Zika virus (ZIKV), a mosquito-borne virus, has caused significant disease in humans during outbreaks over the last decade. Currently, there is no approved preventive vaccine or specific therapeutic drug against ZIKV. The World Health Organization declared a Public Health Emergency of International Concern regarding microcephaly and other neurological disorders caused by ZIKV during pregnancy in 2016, highlighting the importance of understanding the role of the maternal-fetal barrier in this viral disease. The mechanism by which ZIKV causes placental pathogenesis, however, remains unclear. In this study, our data elucidate a previously unrecognized mechanism underlying ZIKV infection that causes severe placental inflammation by activating PANoptosis. Furthermore, we propose a treatment that effectively inhibits ZIKV-induced PANoptosis and attenuates the inflammatory response in trophoblast cells *in vitro* and *in vivo*.

## INTRODUCTION

Zika virus (ZIKV) was first identified in 1947 and circulates between nonhuman primates and *Aedes* mosquitoes in African forests (1). ZIKV belongs to the *Orthoflavivirus* genus within the *Flaviviridae* family and is primarily transmitted by the bite of infected *Aedes* mosquitoes (2, 3). Blood, sexual, and vertical transmission have also been documented in ZIKV spread (4, 5). The outbreak of ZIKV epidemics and the congenital Zika syndrome (CZS) caused by ZIKV infection in 2015 made it a public health emergency of international concern (4, 6). To date, the World Health Organization (WHO) regional office has declared that a total of 92 countries and territories have experienced current or previous Zika virus transmission (7). There is currently no approved preventive vaccine or specific therapeutic drug against ZIKV.

While ZIKV infection generally causes mild symptoms, such as fever, rashes, arthralgia, myalgia, and conjunctivitis (8), maternal infection during pregnancy can lead to severe complications such as an increased risk of premature birth, miscarriage, stillbirth, and CZS. Infants with CZS exhibit microcephaly or other congenital malformations, including brain calcifications, limb contractures, high muscle tone, eye abnormalities, and hearing loss (5, 9–11). Transmission of ZIKV from mother to fetus occurs in all trimesters of pregnancy, with the highest risk in the first trimester (12, 13). Clinical evidence has demonstrated the presence of ZIKV in the placenta, amniotic fluid, infant’s blood, cord blood, and cerebrospinal fluid (14). Despite the extensive research on ZIKV in the past decades, the pathogenesis of ZIKV, particularly the mechanisms for its vertical transmission and placental pathological alterations, remains largely unclear.

PANoptosis is a unique modality of programmed cell death (PCD) that can occur during infection and inflammation, which is usually mediated by a complex known as PANoptosome. PANoptosis shares cellular features and molecular mechanisms with apoptosis, pyroptosis, and necroptosis but cannot be accounted for by any of them alone (15–17). Of note, apoptosis proceeds through either an extrinsic or intrinsic pathway and is a caspase-8- or caspase-9-dependent form of cell death, which is activated by executioner caspases such as caspase-3 and caspase-7 (18, 19), whereas pyroptosis is a lytic form of pro-inflammatory cell death that can be initiated by inflammasome activation-mediated caspase-1 cleavage of gasdermin D (GSDMD) (20). Other executioners, such as gasdermin E (GSDME), cleaved by caspase-3, can also activate pyroptosis (21). Another form of programmed cell death, necroptosis, is activated by proteins containing a receptor-interacting protein homotypic interaction motif, including receptor-interacting serine/threonine-protein kinase 1 (RIPK1) and receptor-interacting serine/threonine-protein kinase 3 (RIPK3). Activation of RIPK3 further phosphorylates mixed lineage kinase domain-like (MLKL), the executioner of necroptosis (22). Previous studies have reported that innate immune sensors or regulators can drive the assembly of the PANoptosome to induce PANoptosis, such as Z-conformation nucleic acid binding protein 1 (ZBP1), absent in melanoma 2 (AIM2), transforming growth factor-β-activated kinase 1 (TAK1), and RIPK1 (23). PANoptosis has been demonstrated to be involved in various infectious and inflammatory diseases and can be induced by diverse triggering factors ranging from viruses to fungi, acting as an important host immune defense against pathogen infection (17, 24, 25). Notably, its excessive activation causes severe inflammatory pathological damage to the host. For example, influenza A virus (IAV) infection activates ZBP1-mediated PANoptosis and induces inflammatory damage in the lungs while limiting IAV replication (24, 26–28). Similarly, SARS-CoV-2 infection induces the expression of pro-inflammatory cytokines tumor necrosis factor alpha (TNF-α) and gamma interferon (IFN-γ), which in turn activate PANoptosis, leading to a prolonged and sustained cytokine storm, resulting in severe lung inflammation and pathological damage (29). It is noteworthy that placentas infected by ZIKV develop severe inflammatory response and cell death (9, 30), prompting us to further ask whether PANoptosis plays a role in the process and consequently leads to ZIKV-related placental diseases.

In this study, our data show that ZIKV infection induces PANoptosis and inflammation in trophoblast cells and placentas. The combined use of a pan-caspase inhibitor Z-VAD-FMK with the RIPK3 inhibitor GSK872 or with the RIG-I inhibitor RIG012 can effectively inhibit ZIKV-induced PANoptosis and attenuate the inflammatory response in trophoblast cells *in vitro* as well as *in vivo*.

## RESULTS

### ZIKV replicates in the placenta and causes severe pathological changes

**Fig 1:**
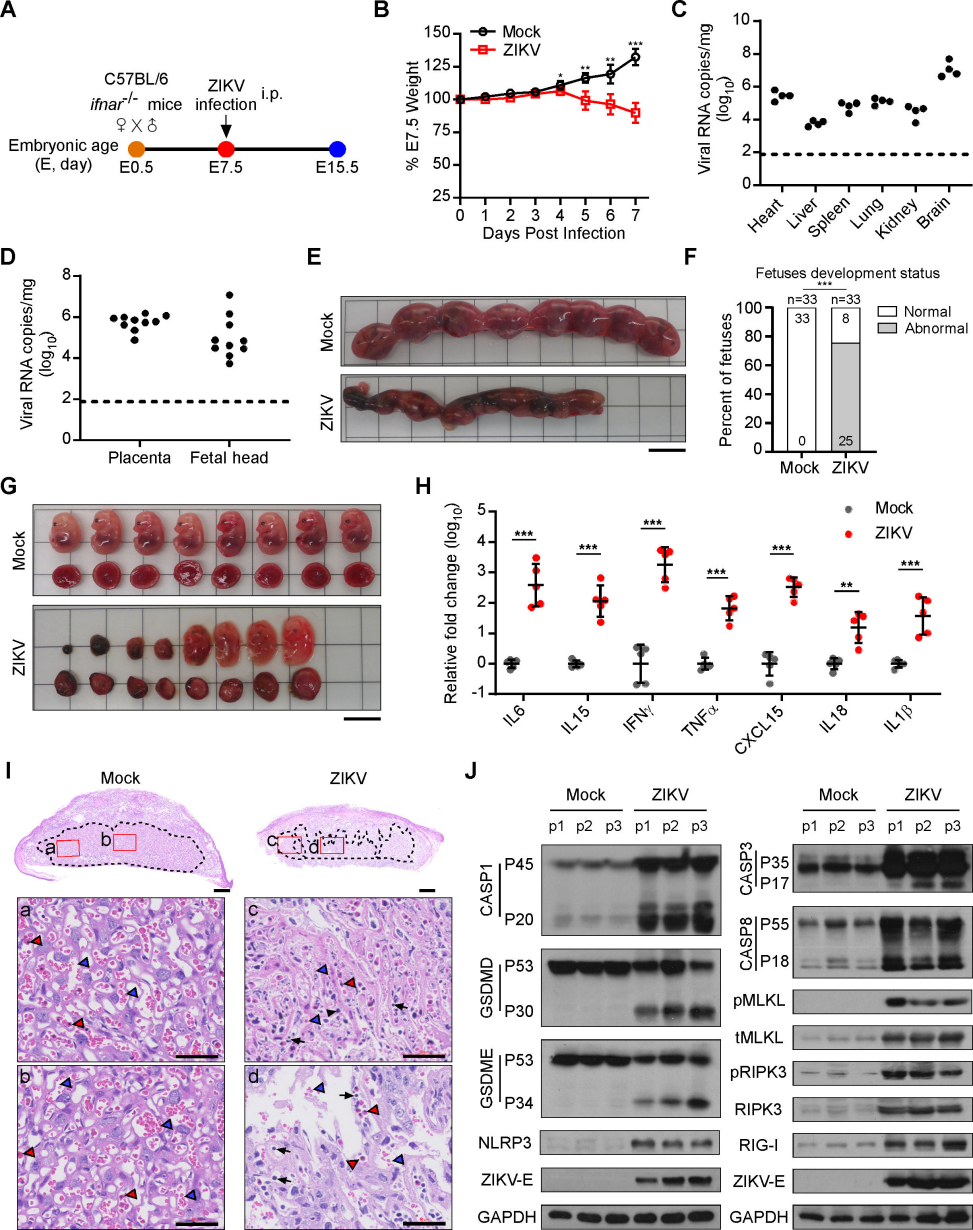
ZIKV infection leads to severe placental pathological changes and inflammation in pregnant dams. *Ifnar1*^−/−^ C57BL/6 dams were intraperitoneally challenged with 1 × 10^5^ PFUs of ZIKV or an equal volume of Vero cell culture supernatant at E7.5 of pregnancy. (**A**) Scheme of the pregnant dams infected with ZIKV *in vivo*. (**B**) Body weight curves of mock- and ZIKV-infected pregnant dams. Data are presented as mean ± SD, Student’s *t* test, *P < 0.05, **P < 0.01, and ***P < 0.001. (**C**) ZIKV RNA in pregnant dams’ specimens was quantified by qRT-PCR (*n* = 4). Dotted line depicts the limit of detection for the assays. (**D**) ZIKV RNA in placenta and fetal head specimens was quantified by qRT-PCR (*n* = 10). Dotted line depicts the limit of detection for the assays. (**E**) Representative morphology of uterus from mock- and ZIKV-infected dams (E15.5). Bar: 1 cm. (**F**) Percentage of normally and abnormally developing embryos from mock- and ZIKV-infected dams. Numbers on bars indicate a normal embryo (top) or an abnormal embryo (bottom). (***P < 0.001 for the distribution of embryo outcomes compared with the mock-infected group by Chi-square test with Yates’ correction) (**G**) Representative images of placentas and fetuses obtained from mock- and ZIKV-infected dams (E15.5). Bar: 1 cm. (**H**) Relative expression levels of pro-inflammatory cytokines were examined using qRT-PCR in placenta specimens of dams (*n* = 5). Data are presented as mean ± SD, Student’s *t* test, **P < 0.01 and ***P < 0.001. (**I**) Representative histological images of the placentas (H&E staining). Black arrowheads: monocytes; red arrowheads: nucleated fetal red blood cells; blue arrowheads: maternal red blood cells (scale bar, 500 µm [left], 50 µm [right]). (**J**) The expression levels of key proteins associated with PANoptosis in murine placental tissues with ZIKV or mock infection were examined by immunoblotting analysis.

Type I interferon receptor-deficient (*Ifnar1*^-/-^) mice, a commonly used animal model for pathogenic viruses, including ZIKV, were employed in this study. To investigate the pathogenesis of ZIKV in the placenta and fetal demise during pregnancy, *Ifnar1*^-/-^ dams with a C57BL/6 background were inoculated intraperitoneally with ZIKV (1 × 10^5^ plaque-forming units [PFUs]) at embryonic day 7.5 (E7.5) and euthanized at E15.5 (Fig. 1A), as previously described (31). The experiment showed that ZIKV infection resulted in lower body weight in pregnant dams compared with the mock controls from 4 to 7 days post-infection (dpi) (Fig. 1B), and viral loads in maternal organs and fetal heads were quantified using a quantitative real-time RT-PCR (qRT-PCR) assay. The results showed that ZIKV RNA was detected in all examined tissues, with the highest copy numbers of viral RNA in placentas and fetal heads (Fig. 1C and D). The pregnant dams were sacrificed, and embryo resorption in uterine horns after ZIKV infection was observed in contrast to mock-infected controls (Fig. 1E). Further separating the fetuses and placentas from the uterine horns, we found that 76% of the fetuses obtained from ZIKV-infected dams exhibited abnormalities, indicating a significant uterine growth restriction (Fig. 1F and G). Placentas from ZIKV-infected dams also showed abnormal morphology and tissue necrosis when compared with the mock-infected controls (Fig. 1G), suggesting that the observed abnormalities of the placenta and fetus were closely associated with ZIKV infection.

We next sought to determine whether inflammation was present in the placentas upon ZIKV infection. Using a qRT-PCR assay, high expression levels of pro-inflammatory cytokine genes, including *IL6*, *IL15*, *IFNγ*, *TNFα*, *CXCL15*, *IL18,* and *IL1β,* were confirmed in ZIKV-infected placentas, suggesting that ZIKV infection may trigger placental inflammation (Fig. 1H). Further histopathological analysis showed that ZIKV-infected placentas were smaller than mock-infected placentas (Fig. 1I), consistent with previously reported cases of congenital ZIKV infection (32). The results also showed that the area of the labyrinthine layer in mouse placentas, which is believed to be responsible for mother-fetus nutrient and gas exchanges, was particularly reduced after ZIKV infection (Fig. 1I). In addition, ZIKV infection also led to uneven distribution of blood vessels, smaller vessel lumen, and tissue hemorrhage, partially in the labyrinthine layer. Furthermore, large numbers of monocytes infiltrated the labyrinthine layer (Fig. 1I). To assess whether cell death was caused by ZIKV infection in placental tissue, we further examined the activation of molecules key to various forms of programmed cell death, such as caspase-1, GSDMD, GSDME, and NLRP3 for pyroptosis, caspase-3 and caspase-8 for apoptosis, and MLKL phosphorylation and RIPK3 phosphorylation for necroptosis activation (33). Our results revealed that all these molecular markers, i.e., cleaved caspase-1, cleaved-GSDMD, cleaved-GSDME, cleaved-caspase-3, cleaved-caspase-8, NLRP3, phosphorylation of MLKL, phosphorylation of RIPK3, and RIG-I, were significantly increased following ZIKV infection, suggesting that ZIKV might have caused PANoptosis activation in the placentas (Fig. 1J).

### ZIKV infection induces PANoptosis in trophoblast cells

**Fig 2:**
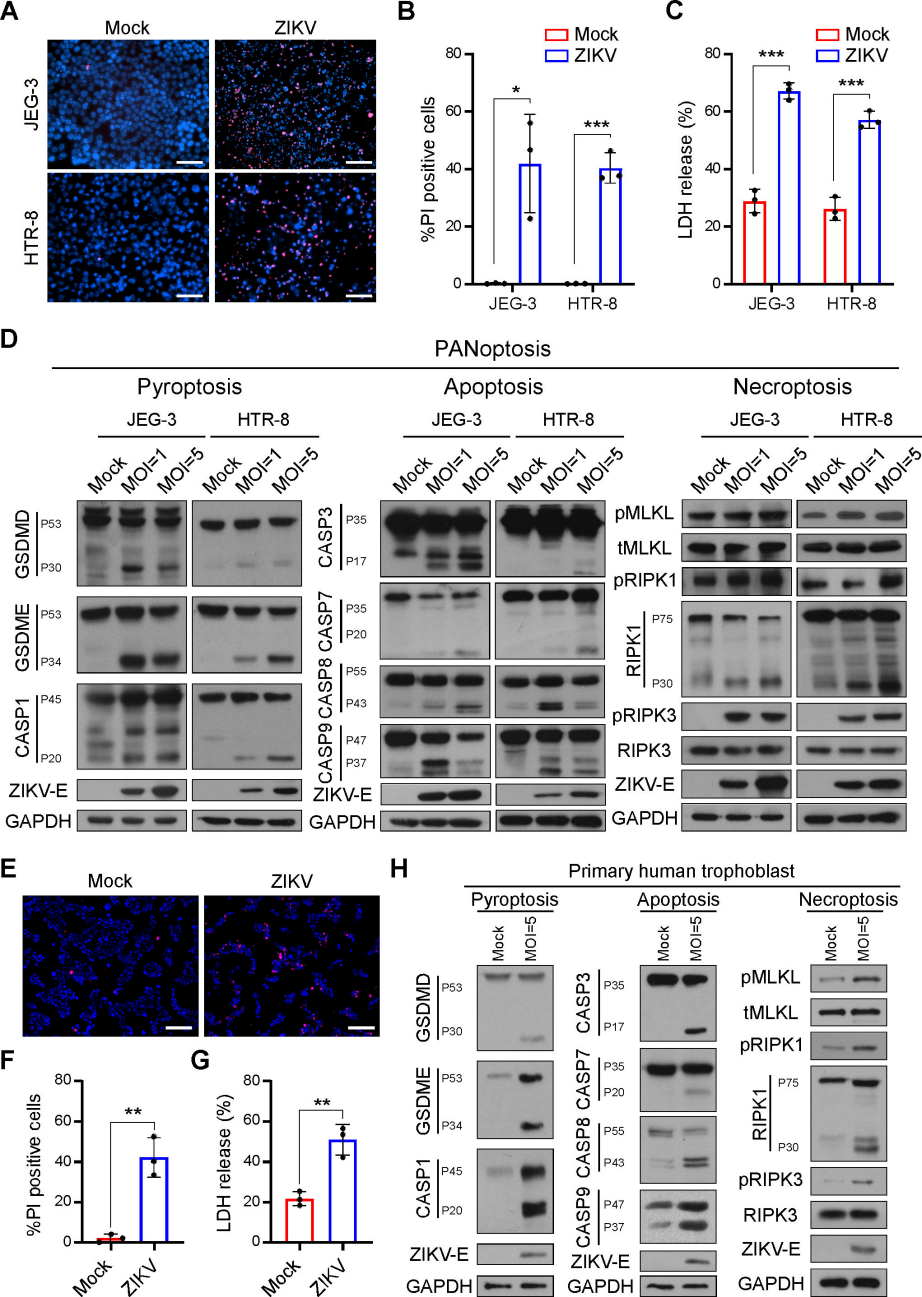
ZIKV infection induces PANoptosis in trophoblast cells. (**A**) Representative micrographs of PI and Hoechst 33342 staining of trophoblast cells with ZIKV (MOI = 5, 48 hpi) or mock infection (scale bar, 100 µm). (**B**) Percentage of PI-positive cells relative to mock infection is shown in the histogram. (**C**) LDH release was determined in supernatant derived from mock- and ZIKV-infected cells in JEG-3 or HTR-8 (MOI = 5, 48 hpi). (**D**) The expression levels of PANoptosis-associated proteins were examined by immunoblotting analysis (48 hpi). (**E**) Representative micrographs of PI and Hoechst 33342 staining of primary human trophoblast (scale bar, 200 µm). (**F**) Percentage of PI-positive cells in primary human trophoblast following mock or ZIKV infection is shown in the histogram (MOI = 5, 48 hpi). (**G**) Comparative analysis of LDH release between mock- and ZIKV-infected primary human trophoblast (MOI = 5, 48 hpi). (**H**) The expression levels of PANoptosis-associated proteins in primary human trophoblast were examined by immunoblotting analysis (MOI = 5, 48 hpi). All data are presented as mean ± SD, Student’s *t* test, *P < 0.05, **P < 0.01, and ***P < 0.001.

The above *in vivo* data prompted us to further examine and characterize ZIKV-induced PANoptosis in cultured human placental trophoblast cells *in vitro*. Human trophoblast cell lines JEG-3 and HTR-8 were infected with ZIKV at a multiplicity of infection (MOI) of 5, respectively. Propidium iodide (PI) staining assay showed that the proportions of PI-positive cells significantly elevated after ZIKV infection in both JEG-3 and HTR-8 lines (Fig. 2A and B), and lactate dehydrogenase (LDH) in cellular supernatant also increased after ZIKV infection, together suggesting that the cells underwent cytolytic cell death (Fig. 2C). To further demonstrate the PANoptotic features of ZIKV-induced cytological changes, we assessed the activation of proteins known to be key to pyroptosis, apoptosis, and necroptosis, respectively. As shown in Fig. 2D, elicited the activation of pyroptosis mediators (GSDMD, GSDME, and caspase-1), apoptosis mediators (caspase-8, caspase-9, caspase-3, and caspase-7), and necroptosis mediators (MLKL and RIPK3), robustly suggesting that ZIKV infection can activate almost all key molecules involved in pyroptotic, apoptotic, and necroptotic pathways in trophoblast cells, which are essential for PANoptosis to occur. These results were consistently observed in ZIKV-infected primary human placental trophoblast cells (Fig. 2E through H). Collectively, these findings suggest that ZIKV infection induces PANoptosis in trophoblast cells.

### ZIKV infection promotes interaction among RIG-I, ASC, and caspase-8 to form PANoptosis-mediating PANoptosome

**Fig 3:**
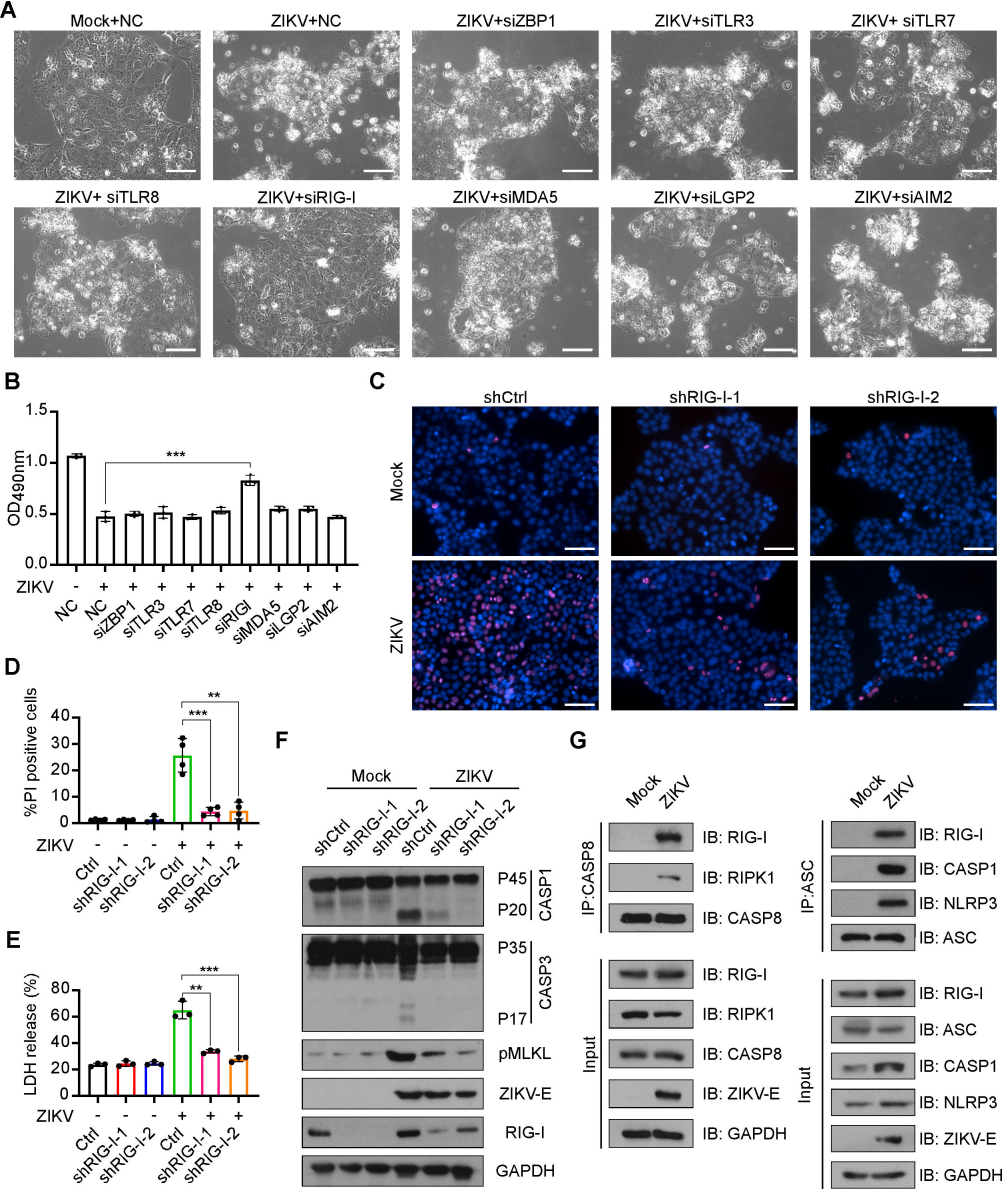
RIG-I interacts with ASC and caspase-8 to form RIG-I PANoptosome mediating PANoptosis activation. (**A**) Representative micrographs of mock- or ZIKV-infected JEG-3 cells (MOI = 5, 48 hpi) transfected with siRNAs targeting ZBP1, TLR3, TLR7, TLR8, RIG-I, MDA5, LGP2, or AIM2, respectively. NC, negative control (scale bar, 100 µm). (**B**) Cell viability of mock- or ZIKV-infected JEG-3 cells (MOI = 5, 48 hpi) transfected with siRNAs targeting ZBP1, TLR3, TLR7, TLR8, RIG-I, MDA5, LGP2, or AIM2, respectively. NC, negative control. (**C**) Representative micrographs of PI and Hoechst 33342 staining of RIG-I knockdown-JEG-3 cells with ZIKV (MOI = 5, 48 hpi) or mock infection (scale bar, 100 µm). (**D**) Percentage of PI-positive cells of RIG-I knockdown-JEG-3 cells with ZIKV (MOI = 5, 48 hpi) or mock infection. (**E**) LDH release was measured in supernatant derived from RIG-I knockdown-JEG-3 cells with ZIKV (MOI = 5, 48 hpi) or mock infection. (**F**) Proteolytic cleavage of caspase-1 and caspase-3, and phosphorylation of MLKL in RIG-I knockdown-JEG-3 cells with ZIKV (MOI = 5, 48 hpi) or mock infection. (**G**) JEG-3 cells were infected with ZIKV at an MOI of 5, and cell lysates were collected at 48 hpi, followed by immunoprecipitation using an anti-caspase-8 antibody or an anti-ASC antibody, respectively, and IgG served as the negative control. The immunocomplexes were analyzed with anti-RIPK1, anti-RIG-I, anti-caspase-8, anti-ASC, anti-caspase-1, and anti-NLRP3 antibodies by western blotting. All data are presented as mean ± SD, one-way ANOVA, **P < 0.01 and ***P < 0.001.

Previous studies have shown that PANoptosis can be initiated by nucleic acid receptors, such as ZBP1 and AIM2, when recognizing exogenous nucleic acid stimuli (34, 35). To identify the initiating receptor for the activation of PANoptosis induced by ZIKV in trophoblast cells, we first employed small-interfering RNA (siRNA) silencing strategy to screen a pool of nucleic acid receptors, including ZBP1, AIM2, TLR3, TLR7, TLR8, RIG-I, MDA5, and LGP2 by evaluating changes in their effects on ZIKV-induced trophoblast death, respectively (Fig. S1). Our results showed that knocking down RIG-I expression inhibited the cell death most potently compared with the siRNA negative control and other nucleic acid receptor groups (Fig. 3A and B) and reduced PI-positive cells and LDH release triggered by ZIKV infection (Fig. 3C through E). Moreover, the activation of PANoptosis mediator proteins, such as caspase-1, caspase-3, and MLKL, could all be suppressed when RIG-I was knocked down, indicating that RIG-I may be a sensing receptor for ZIKV-induced PANoptosis in trophoblasts (Fig. 3F).

To further identify the specific pathway via which RIG-I activates PANoptosis, we performed co-immunoprecipitation assays to test whether RIG-I interacts with key proteins functioning in pyroptotic, apoptotic, and necroptotic pathways. Our results revealed that caspase-8 was coprecipitated with RIG-I and RIPK1 upon ZIKV infection (Fig. 3G), and that ASC physically binds with RIG-I, caspase-1, and NLRP3 (Fig. 3G), suggesting that ZIKV infection could promote interaction among caspase-8, RIPK1, RIG-I, NLRP3, caspase-1, and ASC to form the PANotosome, which is essential for the activation of PANoptosis.

### Z-VAD-FMK and GSK872 treatment inhibits PANoptosis and inflammation in trophoblast cells

**Fig 4:**
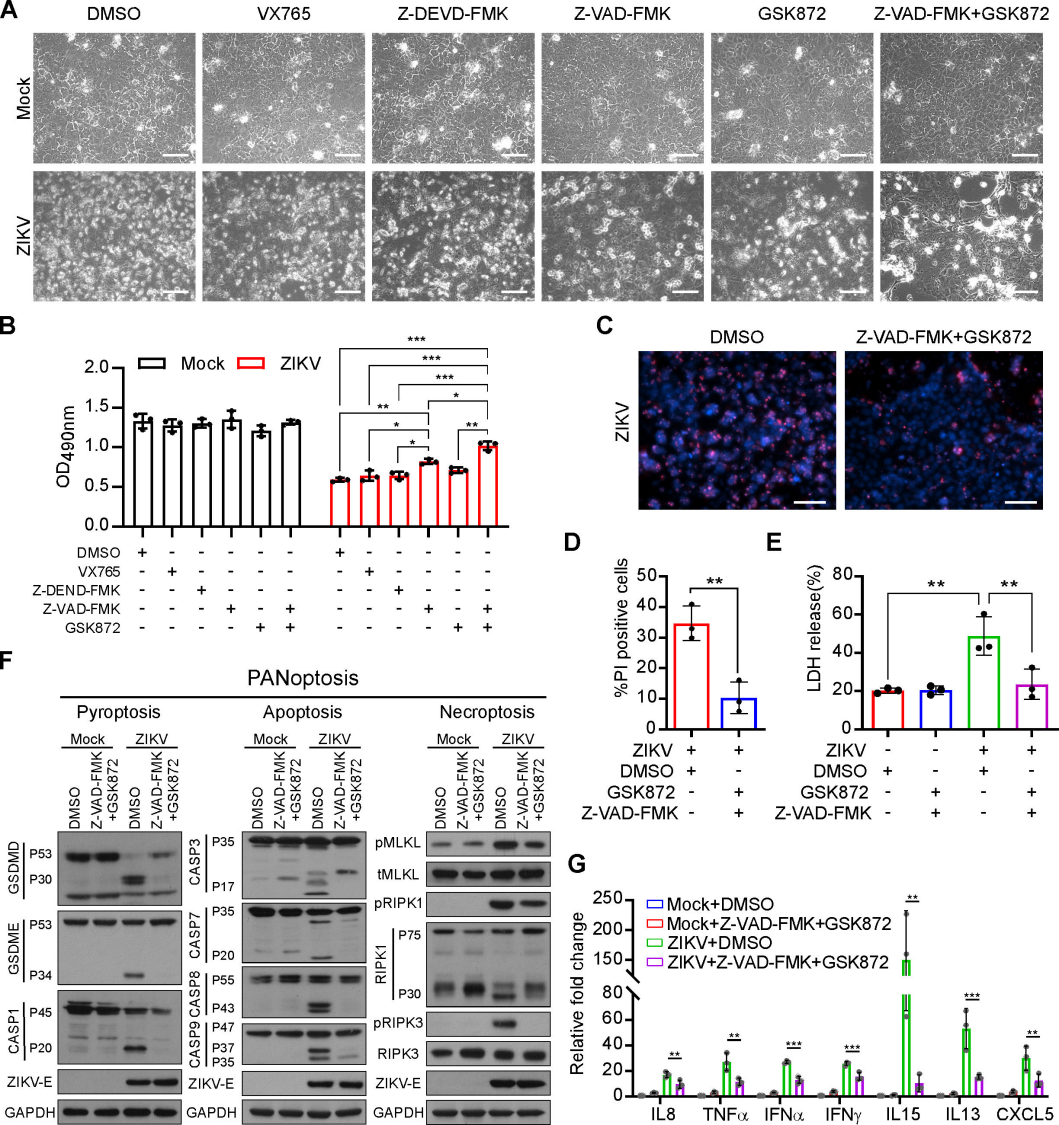
Z-VAD-FMK and GSK872 synergistically inhibit PANoptosis and inflammation. (**A**) Representative micrographs of mock- or ZIKV-infected JEG-3 cells (MOI = 5, 48 hpi) treated with control vehicle, VX765, Z-DEVD-FMK, Z-VAD-FMK, GSK872, or combined treatment of Z-VAD-FMK and GSK872 (scale bar, 100 µm). (**B**) Cell viability of mock- and ZIKV-infected JEG-3 cells (MOI = 5, 48 hpi) treated with control vehicle, VX765, Z-DEVD-FMK, Z-VAD-FMK, GSK872, or combined treatment of Z-VAD-FMK and GSK872. Data are presented as mean ± SD, one-way ANOVA, *P < 0.05, **P < 0.01, and ***P < 0.001. (**C**) Representative images of PI and Hoechst 33342 staining of mock- or ZIKV-infected JEG-3 cells (MOI = 5, 48 hpi) co-treated with Z-VAD-FMK and GSK872 or control vehicle (scale bar, 100 µm). (**D**) Percentage of PI-positive cells of mock- or ZIKV-infected JEG-3 cells (MOI = 5, 48 hpi) co-treated with Z-VAD-FMK and GSK872 or control vehicle. Data are presented as mean ± SD, Student’s *t* test, **P < 0.01. (**E**) LDH release was measured in supernatants derived from mock- or ZIKV-infected JEG-3 cells (MOI = 5, 48 hpi) co-treated with Z-VAD-FMK and GSK872 or control vehicle. Data are presented as mean ± SD, one-way ANOVA, **P < 0.01. (**F**) The expression levels of PANoptosis-associated proteins in mock- or ZIKV-infected JEG-3 cells (MOI = 5, 48 hpi) co-treated with Z-VAD-FMK and GSK872 or control vehicle were examined by immunoblotting analysis. (**G**) Relative expression levels of pro-inflammatory cytokines were determined using qRT-PCR in mock- or ZIKV-infected JEG-3 cells (MOI = 5, 48 hpi) co-treated with Z-VAD-FMK and GSK872 or control vehicle. Data are presented as mean ± SD, one-way ANOVA, *P < 0.05 and **P < 0.01.

Next, we investigated whether PANoptosis can be a potential therapeutic target for placental pathogenic inflammation. Several inhibitors known to suppress key proteins of the PANoptosis pathway were tested for their therapeutic effects in trophoblast cells, among which were included selective caspase 1 inhibitor VX765 (10 µM), caspase 3 inhibitor Z-DEVD-FMK (30 µM), pan caspase inhibitor Z-VAD-FMK (40 µM), and specific inhibitor of RIPK3 phosphorylation GSK872 (3 µM). The above inhibitors were applied to treat ZIKV-infected JEG-3 cells, and the results showed that co-treatment with Z-VAD-FMK (40 µM) and GSK872 (3 µM) could significantly inhibit cell death triggered by ZIKV infection. The inhibitory effect was far more potent than those of separate treatments with VX765, Z-DEVD-FMK, Z-VAD-FMK, or GSK872 alone (Fig. 4A and B). In addition, Z-VAD-FMK and GSK872 co-treatment remarkably reduced the number of PI-positive cells and LDH release induced by ZIKV infection (Fig. 4C through E). Furthermore, activation of molecules key to the induction of pyroptosis, apoptosis, or necroptosis was robustly inhibited after the Z-VAD-FMK and GSK872 co-treatment, indicating that PANoptosis is repressed by Z-VAD-FMK and GSK872 (Fig. 4F). Moreover, the mRNA expression levels of pro-inflammatory cytokines, such as *IL8*, *IL1β*, *TNFα*, *IFNα*, *IFNγ*, *IL15*, *IL13*, and *CXCL5,* were decreased by the combined treatment of Z-VAD-FMK and GSK872 (Fig. 4G). Together, our data suggest that ZIKV-induced PANoptosis and inflammation in trophoblast cells could be antagonized by a combination of Z-VAD-FMK and GSK872 inhibitors.

### Z-VAD-FMK and GSK872 treatment attenuates PANoptosis and inflammation in the placentas of pregnant dams

**Fig 5:**
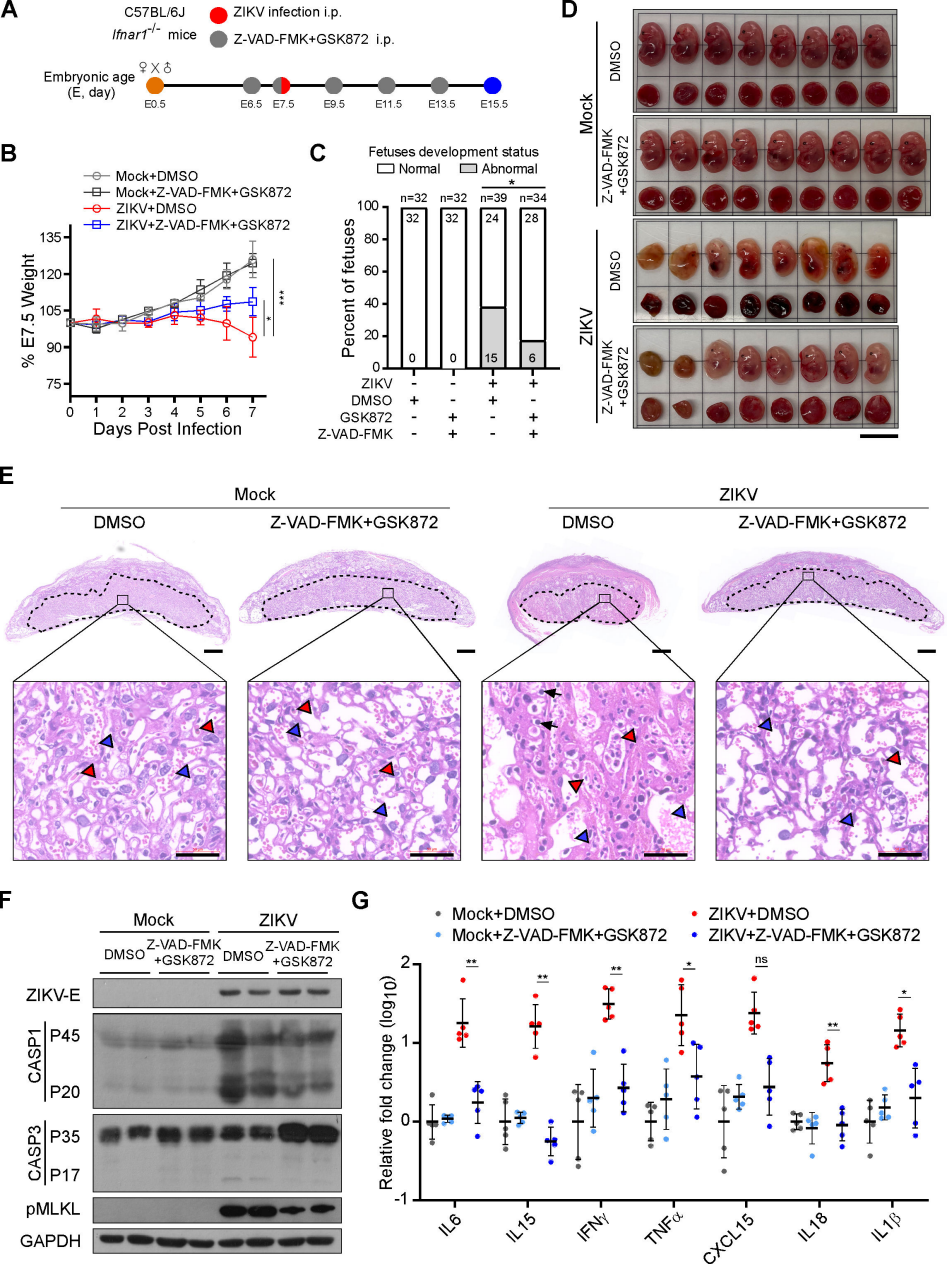
Z-VAD-FMK and GSK872 synergistically alleviate the pathological inflammation in the placentas of pregnant dams. *Ifnar1*^−/−^ C57BL/6 dams were intraperitoneally challenged with 1 × 10^5^ PFUs of ZIKV or an equal volume of Vero cell culture supernatant at E7.5 of pregnancy and co-treated with Z-VAD-FMK (10 mg/kg, i.p.) and GSK872 (10 mg/kg, i.p.) or vehicle (PBS/DMSO) from E6.5 to E13.5 every 2 days and euthanized at E15.5. (**A**) Scheme of treating mock- or ZIKV-infected pregnant dams with Z-VAD-FMK plus GSK872 or vehicle *in vivo*. (**B**) Body weight curves of mock- or ZIKV-infected pregnant dams co-treated with Z-VAD-FMK and GSK872 or vehicle. Data are presented as mean ± SD, one-way ANOVA, *P < 0.05 and ***P < 0.001. (**C**) Percentage of normally and abnormally developing embryos of mock- or ZIKV-infected pregnant dams co-treated with Z-VAD-FMK and GSK872 or vehicle. Numbers on bars indicate a normal embryo (top) or an abnormal embryo (bottom) (*P < 0.05 compared with numbers between indicated groups by Chi-square test). (**D**) Representative images of placentas and fetuses obtained from mock- and ZIKV-infected pregnant dams co-treated with Z-VAD-FMK and GSK872 or vehicle (E15.5). (**E**) Representative histological images of the placentas (H&E staining). Black arrowheads: monocytes; red arrowheads: nucleated fetal red blood cells; and blue arrowheads: maternal red blood cells (scale bar, 500 µm [top], 50 µm [bottom]). (**F**) Proteolytic cleavage of caspase-1 and caspase-3, and the phosphorylation of MLKL in mock- and ZIKV-infected placentas co-treated with Z-VAD-FMK and GSK872 or vehicle were examined by immunoblotting analysis. (**G**) Relative expression levels of pro-inflammatory cytokines were determined using qRT-PCR in specimens of placenta co-treated with Z-VAD-FMK and GSK872 or vehicle (*n* = 5). Data are presented as mean ± SD, one-way ANOVA, **P < 0.01 and ***P < 0.001.

To further look into the *in vivo* therapeutic effects of the Z-VAD-FMK and GSK872 combination in our animal model, pregnant dams intraperitoneally infected with 1 × 10^5^ PFUs of ZIKV or mock-infected were co-treated with Z-VAD-FMK (10 mg/kg of body weight, i.p.) and GSK872 (10 mg/kg of body weight, i.p.). As shown in Fig. 5A and B, when all pregnant dams were sacrificed at E15.5 of pregnancy to obtain placentas and fetuses, ZIKV infection caused significant weight loss in pregnant dams. However, the illness could be attenuated by the combined Z-VAD-FMK/GSK872 treatment. Further analysis of the morphology of the fetuses revealed that while the incidence of abnormality in ZIKV-infected fetuses was 38.46%, pregnant dams treated with the Z-VAD-FMK and GSK872 combination displayed a significantly lower rate (17.64%) of fetal abnormalities (Fig. 5C and D). In addition, histopathological examination of the placentas showed that the pathological changes caused by ZIKV infection, such as uneven distribution of blood vessels, narrowing of vascular space, tissue hemorrhage, and infiltration of monocytes, were alleviated after combined treatment with Z-VAD-FMK and GSK872 (Fig. 5E). Notably, no adverse effects on maternal survival, tissue histology, fetal development, or placental morphology were observed in pregnant dams treated with control vehicle or Z-VAD-FMK and GSK872 without ZIKV infection (Fig. S2A through D; Fig. 5E). Moreover, after the Z-VAD-FMK/GSK872 co-treatment, proteolytic cleavages of both caspase-1 and caspase-3 and the phosphorylation level of MLKL were found to be repressed in the placentas of ZIKV-infected pregnant dams compared to that in the control vehicle group, indicating that the combined Z-VAD-FMK and GSK872 treatment can indeed suppress PANoptosis induced by ZIKV infection *in vivo* (Fig. 5F). Furthermore, we also found that Z-VAD-FMK plus GSK872 treatment abrogated the increase in mRNA levels of *IL6*, *IL15*, *IFNγ*, *TNFα*, *CXCL15*, *IL18,* and *IL1β* in ZIKV-infected placentas (Fig. 5G). Together, the above data suggest that pan-caspase inhibitor Z-VAD-FMK, when used in combination with RIPK3 phosphorylation inhibitor GSK872, suppresses PANoptosis and attenuates inflammatory placental damages in pregnant dams.

### RIG-I inhibitor RIG012 alleviates the placental inflammation caused by ZIKV infection

**Fig 6:**
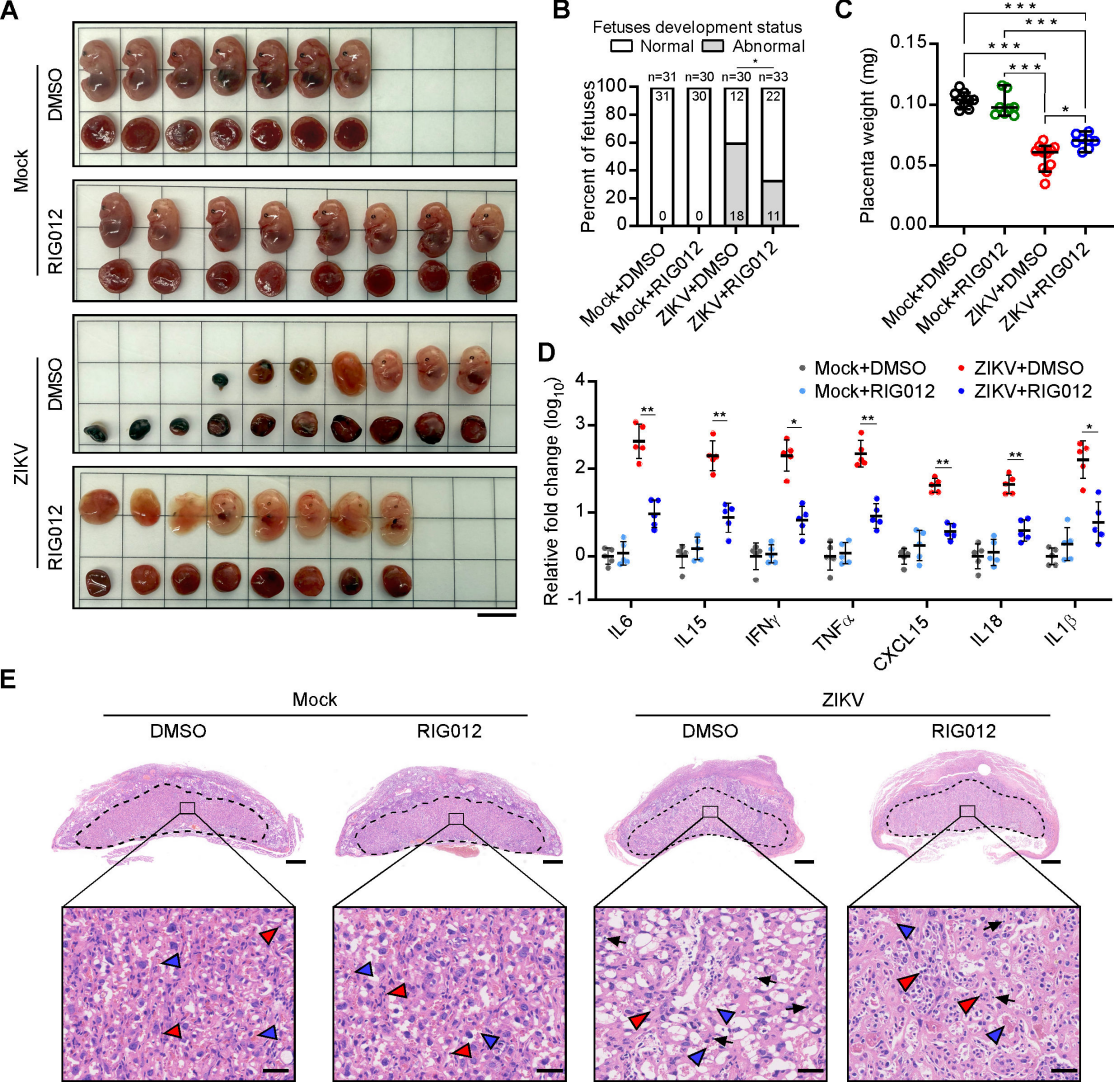
Treatment of pregnant dams with RIG-I inhibitor RIG012 ameliorates ZIKV-induced placental inflammation. ZIKV-challenged *Ifnar1*^−/−^ C57BL/6 dams were treated with RIG012 (10 mg/kg, i.p.) or vehicle control (PBS/DMSO) from E6.5 to E13.5 every 2 days and euthanized at E15.5. (**A**) Representative images of placentas and fetuses obtained from mock- and ZIKV-infected pregnant dams treated with either RIG012 or vehicle (E15.5). (**B**) Percentage of normally and abnormally developing embryos of mock- or ZIKV-infected pregnant dams treated with either RIG012 or vehicle. Numbers on bars indicate a normal embryo (top) or an abnormal embryo (bottom) (*P < 0.05 compared with numbers between indicated groups by Chi-square test). (**C**) Placenta weights of mock- or ZIKV-infected pregnant dams treated with either RIG012 or vehicle. Data are presented as mean ± SD, one-way ANOVA, *P < 0.05 and ***P < 0.001. (**D**) Relative expression levels of pro-inflammatory cytokines were determined using qRT-PCR in placentas treated with either RIG012 or vehicle (*n* = 5). Data are presented as mean ± SD, one-way ANOVA, *P < 0.05 and **P < 0.01. (**E**) Representative histological images of the mock- or ZIKV-infected placentas treated with either RIG012 or vehicle (H&E staining). Black arrowheads: monocytes; red arrowheads: nucleated fetal red blood cells; and blue arrowheads: maternal red blood cells (scale bar, 500 µm [top], 50 µm [bottom]).

As our above experiments identified RIG-I as a key sensor driving ZIKV-induced PANoptosis in trophoblast cells, we next further evaluated the therapeutic potential of RIG-I inhibition using the inhibitor RIG012 in ZIKV-infected *Ifnar1*^-/-^ dams. As shown in Fig. 6A through E, when *Ifnar1*^-/-^ dams were inoculated intraperitoneally with 1 × 10^5^ PFUs of ZIKV at E7.5 and treated with either RIG012 (10 mg/kg, i.p.) or carrier solvent (PBS/DMSO). The RIG012 treatment alleviated the morphological defects in both fetuses and placentas after ZIKV infection (Fig. 6A) and reduced the fetal abnormality rate from 60% to 26.67% (Fig. 6B). Placental weight was also partially restored in RIG012-treated pregnant dams following ZIKV infection (Fig. 6C). In addition, the mRNA levels of *IL6*, *IL15*, *IFNγ*, *TNFα*, *CXCL15*, *IL18,* and *IL1β* in ZIKV-infected placentas were significantly decreased by RIG012 treatment (Fig. 6D). Furthermore, this experimental therapy attenuated the pathological changes caused by ZIKV infection in placentas (Fig. 6E). Of note, no apparent adverse effects on maternal survival, tissue histology, fetal development, or placental morphology were detected in pregnant dams treated with control vehicle or RIG012 without ZIKV infection (Fig. S2A through D; Fig. 6E). Collectively, these data demonstrate that the RIG-I inhibitor RIG012 may ameliorate the pathological and inflammatory effects of the placenta caused by ZIKV infection.

## DISCUSSION

Our present study demonstrated that ZIKV infection induces PANoptosis in placental trophoblast cells, leading to severe placental inflammation. We further elucidated that ZIKV infection drives PANoptosome formation, with RIG-I nucleating the complex by recruiting ASC, caspase-1, NLRP3, caspase-8, and RIPK1 to activate PANoptosis. We also found that inhibition of PANoptosis by treatment with pan-caspase inhibitor Z-VAD-FMK and RIPK3 inhibitor GSK872 together or RIG-I inhibitor RIG012 can attenuate placental inflammatory damages caused by ZIKV infection. These findings reveal a previously unidentified mechanism by which ZIKV infection triggers PANoptosis in trophoblast cells and provide potential therapeutic targets and new treatment options for ZIKV-associated diseases.

During pregnancy, ZIKV can be transmitted across the placenta to the developing fetus, leading to potential teratogenic consequences in the fetus, which may include a wide spectrum of structural anomalies, functional impairments, and clinical sequelae that usually manifest at birth or in early life (36). The fatality rate of infants born with CZS was 10% (37). Evidence of ZIKV infection in placental tissue from one miscarriage case showed heterogeneous chorionic villi with calcification, fibrosis, perivillous fibrin deposition, patchy intervillositis, and focal villitis (9). Chronic villous inflammation, edema, and trophoblastic epithelium lesions were observed in the placental tissue of another miscarriage caused by ZIKV infection (38). These previous findings suggest that ZIKV infection during pregnancy might be able to induce severe pathogenic inflammation in placentas. Interestingly, a recent study reported that ZIKV infection causes adverse fetal outcomes by activating pyroptosis in the placenta (39), implying a hope of using a pyroptosis inhibitor to suppress mother-fetus transmission of ZIKV. Nevertheless, in our preliminary assessment for the therapeutic effect of caspase-1 inhibitor VX765, its inhibition of cell death and inflammation caused by ZIKV infection was suboptimal, suggesting that ZIKV may also induce other forms of inflammation-associated cell death. On such a backdrop, our current study identifies that the placenta infected with ZIKV not only undergoes pyroptosis but also is subject to apoptosis and necroptosis, which meets the characteristics of a highly interconnected inflammatory cell death process known as PANoptosis. We have isolated and cultured human primary trophoblasts and confirmed this phenomenon *in vitro*. Direct validation may be performed in the future using ZIKV-infected placental samples from pregnancies, or further validation could be conducted using human placental tissues or trophoblast-derived organoids. Moreover, only one Asian lineage ZIKV strain was used in the present study; whether other strains, including prototypical African lineages, can similarly induce placental PANoptosis remains to be investigated.

Pathologically, PANoptotic cell death and its associated inflammatory damage in the placenta following ZIKV infection can contribute to intrauterine growth restriction and adverse fetal outcomes, such as reduced crown-rump length, lower fetal weight, and widespread developmental impairments. In our experimental model, pregnant individuals suffering from ZIKV infection frequently exhibited fetal abnormalities, some of which were severe and fatal. Mechanistically, placental inflammation can compromise nutrient transport and oxygen supply and consequently contribute to developmental defects. Notably, neurological abnormalities in the fetus likely arise from both direct viral infection of neural cells and indirect inflammatory effects. In rare cases where fetuses are carried to term, they may exhibit congenital CZS, characterized by microcephaly, intracranial calcifications, and other neurological disorders. In such a context, while our study provides novel insights into the role of PANoptosis in ZIKV-induced placental damage, further study is needed to fully elucidate the long-term neurological consequences of this process. Moreover, inflammation driven by PANoptosis may be intricately linked to immune dysfunctions, including cytokine storms, a breakdown in immune tolerance, and autoimmune disorders in various other viral infections such as those by SARS-CoV-2 (24, 27, 29). Similarly, the success of ZIKV infection is usually associated with a whole variety of dysregulated immune responses for the virus to evade immune suppression, and the development of such immune dysregulation usually contributes to the pathogenesis of viral diseases. Therefore, this form of inflammatory cell death might represent a critical driver of immune dysregulation, which exacerbates disease pathogenesis, and dysregulation of PANoptosis and PANoptosis-mediated inflammation can be causative of other immunopathic conditions. Hence, understanding the mediating mechanisms for ZIKV-associated PANoptosis might facilitate the development of new targeted therapeutic strategies.

Interestingly, our data demonstrate that combining pan-caspase inhibitor Z-VAD-FMK and RIPK3 inhibitor GSK872 effectively suppressed ZIKV-induced PANoptosis and attenuated the inflammatory conditions *in vitro*, with superior efficacy to either agent alone. Consistently, *in vivo* studies confirmed that the dual inhibitor combination alleviates ZIKV-induced placental inflammatory damage. These findings identify PANoptosis as a key driver of ZIKV-induced placental damage and reveal that its pharmacological inhibition—concurrently targeting pyroptosis, apoptosis, and necroptosis—can potentially be a promising therapeutic strategy. This approach provides translatable benefits by mitigating placental inflammation and improving fetal outcomes in affected pregnancies. However, it remains to be determined whether the therapeutic effects of these inhibitors are solely due to PANoptosis inhibition or also involve immune system modulation and off-target effects.

Of particular note, PANoptosis can be achieved by activation of a unique inflammatory PCD pathway during viral infection, which could expose the virus to the immune system and limit virus replication. However, hyperactivation of PANoptosis can also lead to systemic inflammation and pathological conditions (24). PANoptosis activation is mediated by the formation of a protein complex termed PANoptosome, which renders a molecular scaffold that allows for coordinated interactions and activation of three modes of programmed cell death, i.e., pyroptosis, apoptosis, and necroptosis. Thus far, three PANoptosome complexes with different sensors and regulators have been identified, namely, the ZBP1-, AIM2-, and RIPK1-PANoptosome, respectively (15). The ZBP1 PANoptosome has been found to sense the viral ribonucleoprotein complexes of IAV that trigger PANoptosis (27), whereas the AIM2 PANoptosome has been identified as a double-stranded DNA virus sensor to induce PANoptosis (34). Of note, the RIPK1-PANoptosome assembles in the absence or under the inhibition of TAK1 and during *Yersinia* infection (40). In PANoptosome, caspase-8 and ASC are two key scaffold proteins, and caspase-8 has been previously suggested to promote apoptosis and also found to participate in activating pyroptosis by cleaving the GSDM family proteins (19). In addition, ASC is a key scaffold protein involved in the formation of the inflammasome and mediates pyroptosis and has also been reported to be an essential adaptor for the formation of PANoptosome complexes (27, 41). In this study, we uncover that RIG-I, a cytoplasmic dsRNA sensor, can recruit caspase-8, RIPK1, ASC, caspase-1, and NLRP3, forming a more complex PANoptosome to activate PANoptosis following ZIKV infection. Knockdown or inhibition of RIG-I leads to suppression of PANoptosis and inflammatory cytokine release, suggesting that RIG-I may be crucial for ZIKV-induced inflammation. Nevertheless, it remains unclear how RIG-I functions to mediate the ZIKV activation of PANoptosome, and future studies are warranted to determine whether other RNA viruses can also activate RIG-I and form PANoptosome to induce PANoptosis. Furthermore, while our study demostrates activation of all three programmed cell death pathways—pyroptosis, apoptosis, and necroptosis—during ZIKV infection, the precise roles and relative contributions of each to PANoptosis remain to be fully delineated.

Given that RIG-I is a cytosolic RNA sensor, it is conceivable that the ZIKV genomic RNA can be recognized by RIG-I within the cell. A key objective is therefore to determine whether ZIKV triggers PANoptosis through this RIG-I-dependent recognition. Alternatively, the potential role of ZIKV-encoded proteins in modulating RIG-I to induce PANoptosis also merits exploration. On the other hand, RIG-I is important in innate (and adaptive) immunity; it recognizes viral RNA within the host cells and activates downstream proteins such as MAVS and TBK1, leading to the production of type I IFN and eliciting the innate immune response. Our *in vivo* data show that ZIKV infection actually induces PANoptosis in the placenta of *Ifnar1*^-/-^ C57BL/6 dams, which lack a functional type I IFN signaling pathway. Although further validation using genome editing or pharmacological intervention is warranted, it indirectly indicates that RIG-I promotes ZIKV-induced PANoptosis not through the canonical type I IFN signaling pathway. Besides, our present data show that RIG-I inhibition effectively suppresses PANoptosis in the placenta caused by ZIKV infection *in vivo*. Although these preclinical findings are encouraging, further studies are required to evaluate the clinical translational potential of RIG012 or its analogues.

## MATERIALS AND METHODS

### Cell culture and virus

JEG-3 cells (TCHu195, the Cell Bank of the Chinese Academy of Sciences, Shanghai, China), HEK293T cells, and Vero cells were maintained at 37°C with 5% CO_2_ in DMEM (Gibco, Grand Island, NY, USA) supplemented with 10% fetal bovine serum (FBS) (Gibco, Grand Island, NY, USA), 10 mM L-glutamine (HyClone, Logan, UT, USA), 100 µg/mL streptomycin, and 100 U/mL penicillin. HTR-8/SVneo cells (CRL-3271, ATCC, Manassas, VA, USA) were maintained at 37°C with 5% CO_2_ in RPMI-1640 (Gibco, Grand Island, NY, USA) supplemented with 10% FBS, 100 µg/mL streptomycin, and 100 U/mL penicillin. All cell lines were tested negative for mycoplasma contamination. The Asian lineage ZIKV SZ01 strain (GenBank accession no. KU866423) was propagated in Vero cells. Virus stocks were titrated by plaque assays on Vero cells.

### *In vitro* ZIKV infection of trophoblast cells

Human primary trophoblast cells were isolated from healthy term placentas according to a previously described method (42). Human primary trophoblast cells or JEG-3 cells were seeded in 6-well plates at 5 × 10^5^ cells per well. After 24 h, the cells were washed with PBS and then incubated with ZIKV at the indicated MOI for 2 h at 37°C. Subsequently, the inoculum was removed, and cells were washed with PBS and then replaced with fresh medium. Mock-infected cells were incubated with the culture supernatant from uninfected Vero cells. For inhibitor treatment experiments, cells were incubated with ZIKV in the presence of the indicated inhibitors for 2 h at 37°C, washed once with PBS, and maintained in fresh medium supplemented with the corresponding inhibitors. At designated time points after treatment and ZIKV challenge, cellular proteins or RNA were harvested for subsequent analysis according to experimental requirements.

### ZIKV-induced placental inflammation model

*Ifnar1*^-/-^ C57BL/6 female and male mice (8–12 weeks old) were mated overnight at a 2:1 ratio. The following morning, female mice with a viscous vaginal plug were marked as E0.5 of pregnancy. Pregnant dams were randomly divided into two groups and then challenged with 1 × 10^5^ PFUs of ZIKV or an equal volume of Vero cell culture supernatant by intraperitoneal injection at E7.5 of pregnancy, respectively. The weight and behavior of pregnant dams were recorded daily until E15.5. To evaluate the therapeutic effect of combined Z-VAD-FMK and GSK872 or RIG012, pregnant dams were intraperitoneally pretreated with combined Z-VAD-FMK (10 mg/kg) and GSK872 (10 mg/kg) or RIG012 (10 mg/kg) diluted in PBS/DMSO at E6.5 before infection. Pregnant dams were then intraperitoneally inoculated with 1 × 10^5^ PFUs of ZIKV at E7.5 of pregnancy. Two hours after infection, mice were intraperitoneally injected with Z-VAD-FMK/GSK872 or RIG012, with subsequent maintenance doses administered every 2 days from E7.5 through E13.5. Vehicle-treated mice that received injections of PBS/DMSO served as a control. All pregnant dams were sacrificed at E15.5 of pregnancy, and both maternal and fetal tissues were collected for subsequent molecular analyses and histopathological examination.

### Histopathological evaluation of placenta using hematoxylin and eosin staining

Placenta tissues were fixed in 4% paraformaldehyde for 24 h at room temperature, followed by overnight dehydration. Subsequently, the tissues were embedded in paraffin and sectioned using a microtome. Tissue sections were rehydrated and stained with Mayer’s hematoxylin and ethanolic eosin. Following dehydration, the hematoxylin and eosin (H&E)-stained sections were microscopically examined for histologic changes. Images were obtained using an AxioScan.Z1 and analyzed with the ZEN software (Carl Zeiss MicroImaging).

### Assessment of cell death by propidium iodide staining

Cultured cells on coverslip were washed twice using PBS and stained with 5 µg/mL Hoechst 33342 (Beyotime, Shanghai, China) at the ratio of 1:1,000 at 37°C in dark for 10 min and then restained in PI (Beyotime, Shanghai, China) staining solution (1.5 µM in PBS) at 4°C in dark for 30 min. The stained coverslip was rinsed briefly with PBS to remove unbound dye and subsequently dried to mount in an antifade reagent. Images were obtained using a Zeiss Axio Observer Z1 microscope (Carl Zeiss MicroImaging). PI- and Hoechst-positive cells were analyzed by ImageJ software.

### Lactate dehydrogenase release assay

LDH released into cell culture supernatants was measured using the CytoTox 96 Non-Radioactive Cytotoxicity Assay (Promega) according to the manufacturer’s instructions. Briefly, cells were seeded in a 24-well plate overnight, followed by infection with ZIKV or mock control. Supernatants and cell lysates were collected after 48 hpi and were transferred to a 96-well plate. An equal volume of CytoTox 96 Reagent is added to each well and incubated for 30 min. Stop Solution is added, and the absorbance signal is measured at 490 nm in a plate reader. The LDH activity in the culture supernatant was expressed as a percentage of total LDH in the cell lysates.

### siRNA synthesis and transfection

Control siRNA and gene-specific siRNAs were purchased from RiBoBio (Guangzhou, China). The siRNA was delivered into the cells by using Lipofectamine 3000 transfection reagent (Invitrogen) according to the manufacturer’s instructions. The targeting sequences of siRNA for human indicated genes are as follows:

ZBP1-siRNA: GGAACATCATTACAAGACA

TLR3-siRNA: GCACGAATTTGACTGAACT

TLR7-siRNA: GGGTATCAGCGTCTAATAT

TLR8-siRNA: GAACGGAAATCCCGGTATA

RIG-I-siRNA: TAGTAATGCTGGTGTAATT

MDA5-siRNA: GGACAAGCTTCTAGTTAGA

LGP2-siRNA: GGGATCCTGTGGTCATCAA

AIM2-siRNA: GCAACGTGCTGCACCAAAA

### Short hairpin RNA-mediated gene knockdown in trophoblast cells

Two short hairpin RNAs (shRNAs) targeted to RIG-I were cloned into the pLVX-shRNA vector. Approximately 4 µg shRNA plasmid DNA, 2 µg packaging plasmid pMD2.G, and 4 µg pSPAX2 were co-transfected into HEK293T cells using Lipofectamine 3000 transfection reagent (Invitrogen) according to the manufacturer’s instructions. Viral supernatants were collected 48 h post-transfection, filtered through a 0.45 µm filter to remove cell debris, and then inoculated into JEG-3 cells for another 48 h. Stable cell lines were selected via DMEM containing 1.5 µg/mL puromycin (Sigma, St. Louis, MO, USA). Western blotting was employed to verify RIG-I knockdown efficiency. shRNA sequences were as follows: RIG-I-shRNA1: 5′-TAGTAATGCTGGTGTAATT-3′ ; RIG-I-shRNA2: 5′-CCGGCACAGAAGTGTATAT-3′.

### Western blotting analysis

The harvested tissues or cells were lysed with RIPA lysis buffer (Millipore, Bedford, MA, USA) containing a cocktail of protease and phosphatase inhibitors (Sigma, St. Louis, MO, USA). Protein concentrations of cell lysates were determined with a bicinchoninic acid protein assay (Thermo Fisher Scientific, Rochester, NY, USA). Protein samples were separated by sodium dodecyl sulfate-polyacrylamide gel electrophoresis and transferred onto a polyvinylidene difluoride membrane (Roche, Indianapolis, IN, USA). Nonfat milk (5%) in Tris-buffered saline (20 mM Tris-HCl [pH 7.6] and 135 mM NaCl) containing 0.1% Tween 20 was used to block nonspecific antibody binding sites for 1 h at room temperature. Next, the membranes were incubated with the following primary antibodies, respectively, overnight at 4°C: anti-Caspase1/P20/P10 (1:1,000, Proteintech, 22915-1-AP), anti-Caspase-1(p20) (1:1,000, AdipoGen, AG-20B-0042), anti-GSDMD (1:1,000, Abcam, ab210070, ab209845), anti-GSDME (1:1,000, Abcam, ab215191), anti-Caspase3 (1:2,000, Cell Signaling Technology, #9662), anti-Caspase7 (1:2,000, Cell Signaling Technology, #9492), anti-Caspase8 (1:2,000, Cell Signaling Technology, #9746), anti-Caspase8 (1:2,000, ABclonal, A0215), anti-Caspase9 (1:1,000, Cell Signaling Technology, #9502), caspase-9 p10 (H-83) (1:500, Santa Cruz Biotechnology, sc-7885), anti-Phospho-MLKL (1:1,000, Cell Signaling Technology, #37333, Beverly, MA, USA), anti-MLKL (phospho S358) (1:1,000, Abcam, ab187091) , 1:2,000, ab243142RIP (D94C12) XP Rabbit mAb (1:1,000, Cell Signaling Technology, #3493, Cambridge, England), anti-Phospho-RIP3 (1:1,000, Cell Signaling Technology, #93654, #91702), anti-RIP3 (1:1,000, Proteintech, 68786-2-Ig), anti-Phospho-RIPK1 (1:1,000, Proteintech, 66854-1-Ig), anti-ZIKV E (1:2,000, GeneTex Inc., GTX133314), anti-AIM2 (1:2,000, Proteintech, 66902-1-Ig), anti-ZBP1 (1:2,000, Proteintech, 13285-1-AP), anti-RIG-I/DDX58 (1:2,000, Proteintech, 20566-1-AP), anti-LGP2 (1:2,000, Proteintech, 11355-1-AP), anti-TLR8 (1:2,000, Proteintech, 67317-1-AP), anti-TLR3 (1:1,000, ABclonal Technology, A11778), anti-TLR7 (1:1,000, ABclonal Technology, A0991), anti-TLR7 (ABclonal Technology, Wuhan, China), anti-DHX58 (1:1,000, ABclonal Technology, A8257), anti-MAVS (1:1,000, Cell Signaling Technology, #24930), anti-TBK1 (1:1,000, Cell Signaling Technology, #3013), anti-NLRP3 (1:1,000, Cell Signaling Technology, #15101), anti-ASC (1:1,000, Santa Cruz Biotechnology, sc-271054), and anti-GAPDH (1:20,000, Proteintech, 60004-1-Ig). Subsequently, the membranes were incubated with horseradish peroxidase-conjugated secondary antibodies for 1 h at room temperature, and signals were detected by enhanced chemiluminescence using a commercial kit (Tanon, Shanghai, China) according to the manufacturer’s protocols.

### Co-immunoprecipitation assay

JEG-3 cells were infected with ZIKV for 48 h or mock infected, and the cells were lysed with protein lysis buffer (25 mM HEPES, 150 mM NaCl, 1 mM EDTA, 2% glycerol, 1% NP-40, and a cocktail of protease and phosphatase inhibitors). The lysates were incubated with the indicated antibodies or IgG antibody overnight at 4°C. The precipitates were washed five times with wash buffer (20 mM HEPES, 150 mM NaCl, 1 mM EDTA, 1 mM EGTA, 2% glycerol, and 0.1% NP-40) every 5 min. The immunocomplexes were resuspended in sampling buffer and examined by western blotting analysis.

### Quantitative real-time reverse transcription PCR

Total RNA was extracted from cultured cells or tissues with the TRIzol reagent (Invitrogen, Carlsbad, CA, USA) according to the manufacturer’s instructions. First-strand cDNA synthesis was conducted using random hexamer primers, and qPCR was performed by using the ChamQ SYBR qPCR Master Mix (Vazyme, Nanjing, China) or AceQ qPCR probe Master Mix (Vazyme, Nanjing, China). All readings were normalized to the level of glyceraldehyde-3-phosphate dehydrogenase mRNA. The sequences of primer sets used for qRT-PCR are shown in Tables S1 and S2.

### Statistical analysis

Statistical analyses were performed using GraphPad Prism (version 10.1.2). Data are presented as the mean ± standard deviation from at least three independent biological replicates (the specific *n* values are provided in the figure legends). Chi-square test, Student’s *t*-test, and one-way ANOVA with multiple comparisons were used to determine significance level and are indicated in the figure legends. Statistical significance is indicated as follows: *P < 0.05, **P < 0.01, and ***P < 0.001.

## Data Availability

No data sets were generated or analyzed during the current study.

## SUPPLEMENTAL MATERIAL

Supplemental Figures jvi.01759-25-s0001.docx Figures S1 and S2.

Tables S1 and S2 jvi.01759-25-s0002.docx Primers and probes for real-time RT-PCR.

ASM does not own the copyrights to Supplemental Material that may be linked to, or accessed through, an article. The authors have granted ASM a non-exclusive, world-wide license to publish the Supplemental Material files. Please contact the corresponding author directly for reuse.
