# Supplementary material for: ZIKV infection causes placental inflammation through activating PANoptosis
Source: J Virol. 2025 Nov 20;99(12):e01759-25. doi: 10.1128/jvi.01759-25 (PMC12724194; doi:10.1128/jvi.01759-25)
Supplement: Supplemental Figures — Figures S1 and S2. [file jvi.01759-25-s0001.docx]

**Supplemental Figure**

**S1 Fig.**


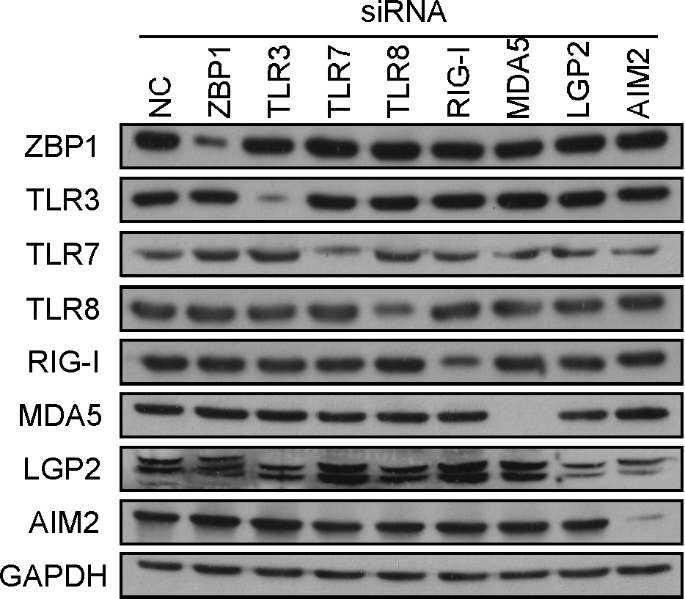


**S1 Fig. Verification of protein expression levels after siRNA transfection in JEG-3 cells.** The expression levels of indicated proteins in JEG-3 cells transfected with siRNAs targeting ZBP1, TLR3, TLR7, TLR8, RIG-I, MDA5, LGP2 or AIM2, respectively, were determined by immunoblotting analysis.

**S2 Fig.**


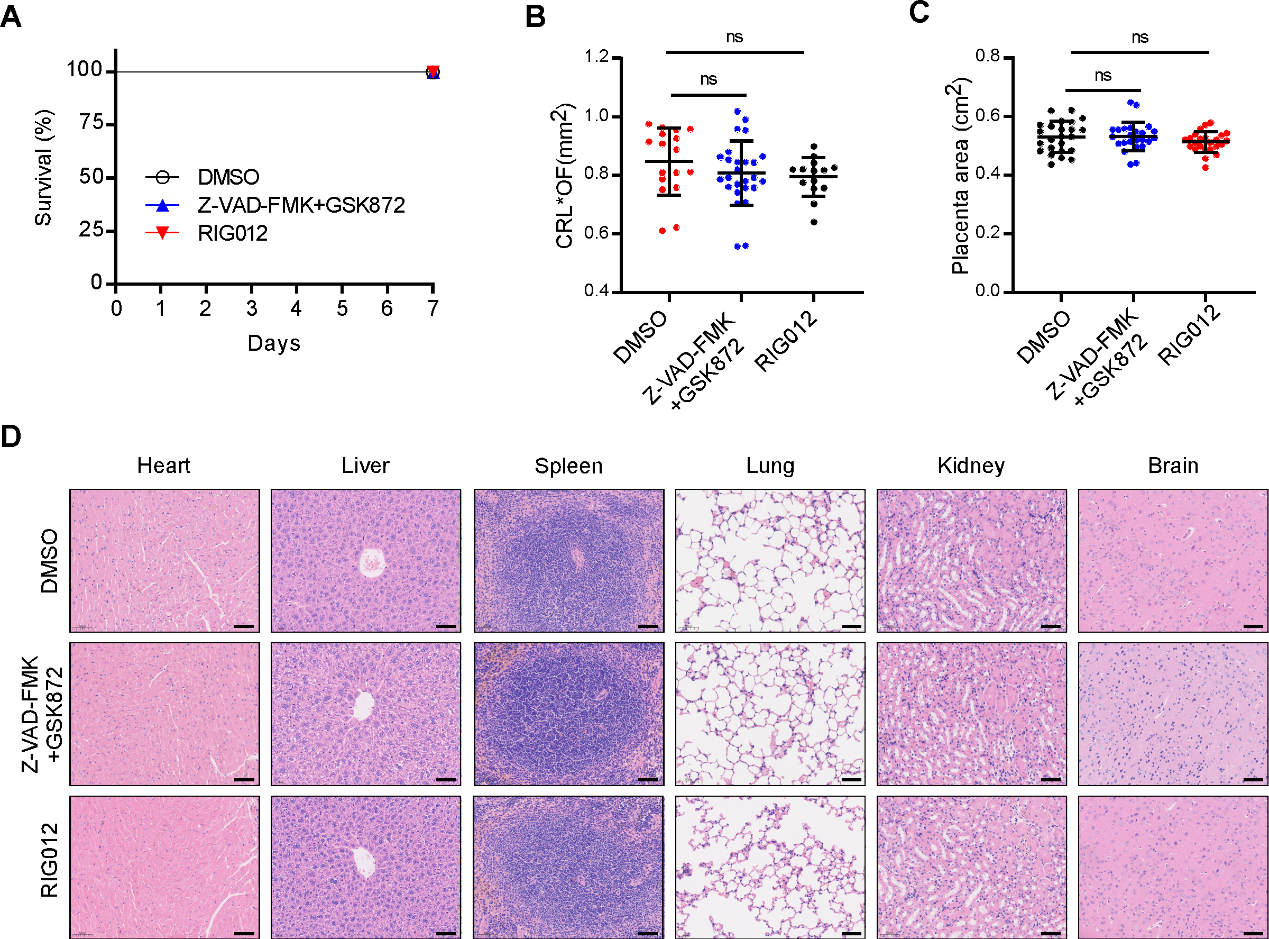


**S2 Fig. Safety Assessment of Inhibitors in** ***Ifnar1*^−/−^ C57BL/6 dams.** *Ifnar1*^−/−^ C57BL/6 dams were administered either a combination of Z-VAD-FMK (10 mg/kg) and GSK872(10 mg/kg), the RIG-I inhibitor RIG012 (10 mg/kg) or an equivalent volume of DMSO (vehicle control) via intraperitoneal injection at embryonic days E6.5, E7.5, E9.5, E11.5, and E13.5, followed by euthanasia at E15.5 for analysis. (A) The survival of *Ifnar1*^−/−^ C57BL/6 dams treated with Z-VAD-FMK/GSK872 or RIG012. Fetus size (B) and placenta area (C) measurement in *Ifnar1*^−/−^ C57BL/6 dams treated with inhibitors or control vehicle without ZIKV infection, fetus size assessed by crown-to-rump length (CRL) times the occipito-frontal (OF) diameter of the head. (D) Representative histology of the tissues from *Ifnar1*^−/−^ C57BL/6 dams (non-ZIKV-infected) treated with inhibitors or control vehicle (H&E staining, scale bar, 50 μm).
