# Supplementary material for: ZIKV infection causes placental inflammation through activating PANoptosis
Source: J Virol. 2025 Nov 20;99(12):e01759-25. doi: 10.1128/jvi.01759-25 (PMC12724194; doi:10.1128/jvi.01759-25)
Supplement: Tables S1 and S2 — Primers and probes for real-time RT-PCR. [file jvi.01759-25-s0002.docx]

**Table S1. Primers for SYBR Green real time RT-PCR**

| Target | Primers | Sequence (5'-3') |
| --- | --- | --- |
| hIL8 | Forward | GCAGCAGGTGAGTGGGCAGT |
|  | Reverse | CTGTACGCCTGGTTCGCTCTGT |
| hIL13 | Forward | CCTCATGGCGCTTTTGTTGAC |
|  | Reverse | TCTGGTTCTGGGTGATGTTGA |
| hIL15 | Forward | TTGGGAACCATAGATTTGTGCAG |
|  | Reverse | GGGTGAACATCACTTTCCGTAT |
| hTNFα | Forward | CCTCTCTCTAATCAGCCCTCTG |
|  | Reverse | GAGGACCTGGGAGTAGATGAG |
| hIFNα | Forward | GCCTCGCCCTTTGCTTTACT |
|  | Reverse | CTGTGGGTCTCAGGGAGATCA |
| hIFNγ | Forward | ACTGAGAGTGATTGAGAGTGGAC |
|  | Reverse | AACCCTCTGCACCCAGTTTTC |
| hCXCL5 | Forward | AGCTGCGTTGCGTTTGTTTAC |
|  | Reverse | TGGCGAACACTTGCAGATTAC |
| hDDX58 | Forward | CTGGACCCTACCTACATCCTG |
|  | Reverse | GGCATCCAAAAAGCCACGG |
| hGAPDH | Forward | AGATCCCTCCAAAATCAAGTGG |
|  | Reverse | GGCAGAGATGATGACCCTTTT |
| mIl6 | Forward | CTGCAAGAGACTTCCATCCAG |
|  | Reverse | AGTGGTATAGACAGGTCTGTTGG |
| mIl15 | Forward | CATCCATCTCGTGCTACTTGTG |
|  | Reverse | GCCTCTGTTTTAGGGAGACCT |
| mIfng | Forward | GCCACGGCACAGTCATTGA |
|  | Reverse | TGCTGATGGCCTGATTGTCTT |
| mTnfa | Forward | CAGGCGGTGCCTATGTCTC |
|  | Reverse | CGATCACCCCGAAGTTCAGTAG |
| mCxcl15 | Forward | TCGAGACCATTTACTGCAACAG |
|  | Reverse | CATTGCCGGTGGAAATTCCTT |
| mIl18 | Forward | GTGAACCCCAGACCAGACTG |
|  | Reverse | CCTGGAACACGTTTCTGAAAGA |
| mIl1b | Forward | GAAATGCCACCTTTTGACAGTG |
|  | Reverse | TGGATGCTCTCATCAGGACAG |
| mGapdh | Forward | AGGTCGGTGTGAACGGATTTG |
|  | Reverse | GGGGTCGTTGATGGCAACA |

**Table S2.** **Primers and probes for Taqman real-time RT-PCR**

| Target | Primers | Sequence (5'-3') |
| --- | --- | --- |
| ZIKV | Forward | CCGCTGCCCAACACAAG |
|  | Reverse | CCACTAACGTTCTTTTGCAGACAT |
|  | Probe | FAM-AGCCTACCTTGACAAGCAGTCAGACACTCAA-BHQ1 |
| hGAPDH | Forward | GACTCATGACCACAGTCCATGC |
|  | Reverse | AGAGGCAGGGATGATGTTCTG |
|  | Probe | FAM-CATCACTGCCACCCAGAAGACTGTG-BHQ1 |
| mGapdh | Forward | TGTGTCCGTCGTGGATCTGA |
|  | Reverse | CCTGCTTCACCACCTTCTTGA |
|  | Probe | FAM-CCGCCTGGAGAAACCTGCCAAGTATG-BHQ1 |
